# Methodological Aspects of Welded Joint Quality Assessment

**DOI:** 10.3390/ma18092148

**Published:** 2025-05-07

**Authors:** Łukasz Muślewski, Michał Pająk

**Affiliations:** 1 Faculty of Mechanical Engineering, Bydgoszcz University of Science and Technology, Al. prof. S. Kaliskiego 7, 85-789 Bydgoszcz, Poland; 2 Faculty of Mechanical Engineering, Casimir Pulaski Radom University, hm. kpt. E. Stasieckiego 54, 26-600 Radom, Poland; m.pajak@urad.edu.pl

**Keywords:** weldability criteria, assessment model, OMS, mathematical modeling, weld quality, fuzzy modeling

## Abstract

The quality of manufacturing processes largely depends on applying modern design methods and technologies. Much progress has been made in the field of metallurgy and the physics of welding, including the weld pool hydrodynamics, the surface and volumetric forces of different origins, the modeling of the SP crystallization process, and the structural transformation morphology in SWC. Additionally, attempts have been made to use the normalized parameters of fracture mechanics to evaluate the material SU. The above-mentioned solutions have also been given a more specific character by establishing SINTAP procedures and computational welding mechanics (CWM). This study discusses a universal method for welded joint evaluation according to the most significant criteria and relevant descriptive features.

## 1. Introduction

The development of welding as a manufacturing technology observed in recent years has led to the construction of new quality structures characterized by high reliability, safety, efficiency, and environment friendliness.

The progress in welding is, among others, the result of the following:

- Development and implementation of high and increased-strength steels and other materials with special physical and mechanical properties accompanied by research into complex issues of weldability;
- Development of new construction solutions with the use of welding methods based on highly concentrated heat sources such as plasma streams, lasers, and electron beams, as well as the mechanization and automation of the welding process;
- Discovery of new phenomena accompanying the processes of welding.

One of the most important issues in welding is the correct weldability assessment. Currently, welding is considered a technological process of special character whose results cannot be fully checked or controlled by production tests. The above statement is consistent with the concept that most defects occur during manufacturing and technological design. Practically, according to the norms, including EN 729, engineering activities focus both on practical tests and theoretical assumptions. They are based on expert systems using computer simulations of physical processes. One of the most significant achievements of contemporary technology is using computational welding mechanics (CWM) [1]. It enables deep knowledge of the technological processes.

Thus, CWM directly impacts the safety and reliability of systems and engineering objects. The prediction and simulation of the desirable periods—the times of the correct operation of structural elements—are the main tasks involved in design, technology, and manufacturing, as they directly affect the operation processes.

## 2. Analysis of the Current State of Knowledge

CWM makes it possible to extend the analysis of the temperature, stress, and strain, including the evolution of the microstructure during the welding process. According to the strain analysis, the deformation caused by the material volumetric strain due to heat expansion and phase transformation generates the main load.

More specifically, CWM accounts for the complex relation of the feedback character, which enables determining the relations between the following modules: the heat field (PT); the microstructure evolution (ME); and the mechanical field (MF). Hence, assessing the relations between the above-mentioned modules should include a dimensional analysis of the PT-ME-MF and a precise recording of their occurrences during processes. A distinct asymmetry between (PT), (ME), and (MF) occurs here, as well. From a broader perspective, OSM also accounts for the computer-aided design process (CAD) and computer-aided manufacturing (CAM) elements. The modules (PT), (MW), and (MF) provide the basis for the construction of algorithms to be used in theoretical and practical welding engineering.

**Figure 1:**
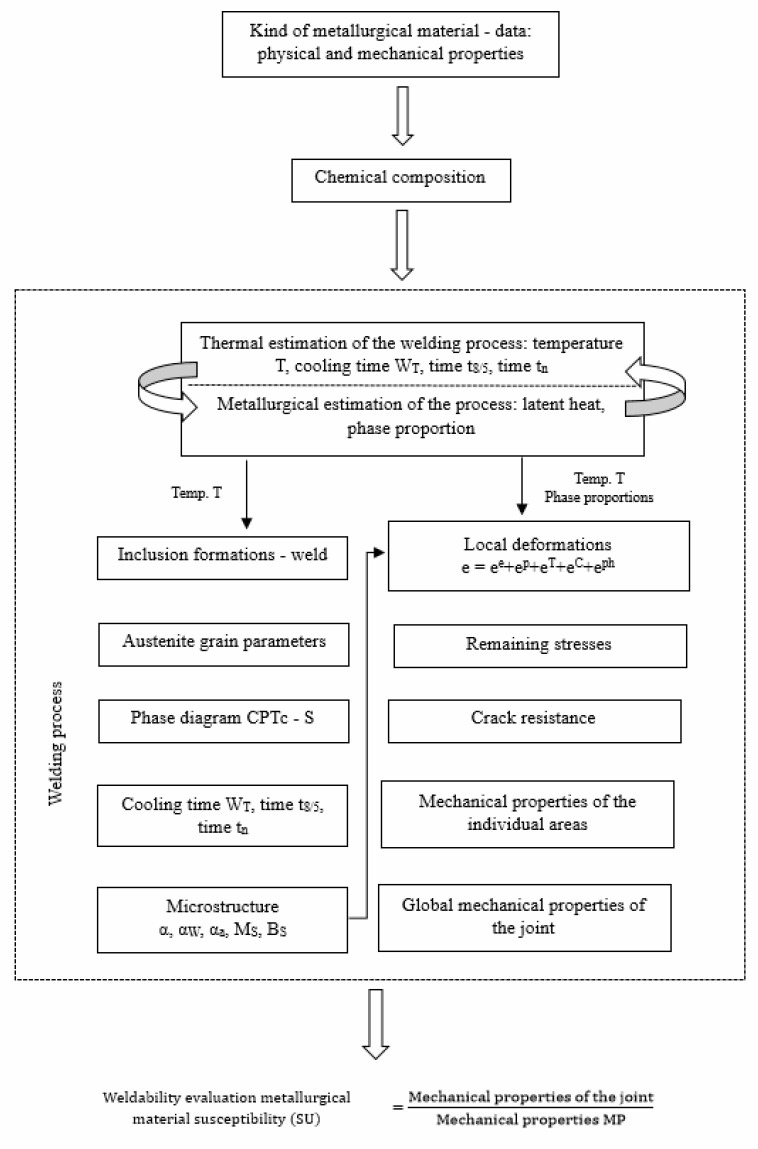
Scheme for the calculations of the microstructure and properties of the SP and HAZ [2], where.

The research methodology is presented in Figure 1 in the form of a block scheme for the calculation of the weld microstructure and properties (WP), as well as the heat-affected zone (HAZ) [2]. Eventually, it leads to a partial assessment of the metallurgical material’s susceptibility to the welding process and the evaluation of its weldability. Such an assessment significantly contributes to the development of the metallurgy and physics of the welding process, including the hydrodynamics of the weld pool and the surface and volumetric forces of different origins, as well as the modeling of the crystallization process SP and the morphology of structural changes in the HAZ.

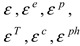
represent the total strain; represent total, elastic, plastic, heat, creeping, and phase changes.

Practically, there have been attempts to use normalized fracture mechanics parameters to assess the material *SU*, e.g., *K_Ith_*/*K_IC_* under the conditions of cold cracking or during operation *δ/δ_c_*, *J/J_c_* [3]. Hence, the following is established:

(1) $${\mathrm{SU}}_{1}=K_{\mathrm{Ith}}{/K}_{\mathrm{IC}}$$

(2) $$SU_{2}=\delta /\delta_{c}\neq J/J_{c}$$

(3) $$SU_{1}\neq SU_{2}$$

where:

*K_Ith_/K_IC_*: stress intensity factor/critical stress intensity factor;
*δ/δ_c_*: energy released during fracture/critical energy released during fracture;
*J/J_c_:* integral value of the parameter/limit value of the parameter.

This implies that one parameter globally describes the degree of material susceptibility to the welding process, which undoubtedly complicates an assessment of the materials’ weldability at the manufacturing stage and during welding. The mechanical properties are formed as early as the initial stage of the weld crystallization.

In such a case, while cooling the weld pool at a temperature of 2300 ÷ 800 K, melted oxygen and deoxidizers react in the liquid of the weld pool, forming complex oxide inclusions with dimensions from 0.1 to 1 μm. In the range of temperatures 1800 ÷ 1600 K, the crystallization of liquid into ferrite δ begins and evolves with the effect of the oxide inclusions. After the total transformation of ferrite δ into austenite γ at a temperature of 1100 ÷ 500 K, austenite γ transforms into different forms of ferrite: initial, Widmanstätten, and coniferous ferrite [4]. There is no comprehensive model that accounts for all these processes. Thus, the welded joint structure modeling process requires the acceptance of a macroscopic scale for the physical description of heat transport and fluid flow and a microscopic scale of grain nucleation and its hyperplasia growth.

The scheme presented in Figure 1 shows that the assessment of the welding process is coupled with a metallurgic transformation, from inclusions to the transformation of the microstructure, though of heterogenic character. Depending on the nature of the process, it is difficult to provide single high-quality welded joints. Basically, welded joints have more defects than the surrounding parent material. Currently, it is believed that, at the initial stage, the microscopic crack mechanism is connected to the formation of discontinuities in the form of caves (holes), their growth, and connecting. It is also assumed that the caves occur during inclusions and are cylindrical. In welded joints, this role is played mainly by oxides, sulfides, and carbides. The stresses in the vicinity of discontinuities can be determined from the following dependence (4) [5]:

(4) $$\sigma =\frac{K}{\sqrt{\pi \left(r+\delta \right)+\sqrt{\pi \;r}}}\sqrt{\frac{\sqrt{1-\left(v^{2}/c_{d}^{2}\right)\sin^{2}\Theta \;}+\cos\;\Theta }{1-\left(v^{2}/c_{d}^{2}\right)\sin^{2}\Theta }},$$

where:

*K*: stress intensity factor;
*v*: crack tip propagation velocity;
*c_d_*: velocity of vortex-free wave propagation;
*r*: radius;
*Θ*: polar coordinates with a beginning situated in the crack tip.

(5) $$\delta =\frac{\kappa \;K^{2}}{\sigma_{o}^{2}}$$

(5) is introduced to (4) to remove the peculiarities. For instance, if *κ* = 0.025, then the following is established:

(6) $$\sigma_{\max}=\frac{\sigma_{o}}{\sqrt{0.025\;\pi }}=3.6\;\sigma_{o}.$$

The growth of the radius of the cylindrical opening (*a*) occurs according to the dependence:

(7) $$\frac{1}{a}\;\frac{da}{dt}=\beta {\left(\frac{\sigma }{\sigma_{o}}-1\right)}^{n};\;\;\;\;\;\sigma >\sigma_{o}$$

where:

*a*: radius of cylindrical opening;
*t:* time;
*β*: constant (fluidity parameter);
*σ_o_*: constant stress.

Openings are formed near the *i*-th particles when the stresses exceed some critical value *σ*^(*i*)^*_el_*, which depends on the particle size:

(8) $$\sigma_{el}^{\left(i\right)}=sof\;\sigma_{o}\left[1+{\left(\frac{b_{o}}{a_{i}}\right)}^{1/2}\right]$$

where:

*a_i_*: radius of the particle;
*b_o_*: the standard value of particle radius;
*sof*: coefficient characterizing the particle wrap combination.

Dependences (4)–(8) provide the mathematical characteristics of a model illustrating the microscopic process of the material deformation near the crack tip [5,6], including particles–nonmetallic inclusions. In practice, to obtain comprehensive deformation information, one should perform the following: use dependence (4) to calculate σ and convert it to ε in the elastic regime; integrate dependence (7) to determine a(t) and then relate the void growth to the plastic strain, e.g., via porosity models; and account for the material properties (e.g., *E*, *σ*_0_) and the distribution of particles in the material.

In studies [2,4], this problem is presented in a slightly different way, with a focus on the influence of heterogeneity on the parameters that characterize the impact of mechanical joints on the assessment of crack resistance with reference to classic solutions of fracture mechanics.

In addition, test results suggest that the most important achievements in evaluating cracking include effective CWM simulations and fatigue models based on multiple-variant decisions. For instance, one study [7] showed how numerical simulations can predict residual stresses in large welded structures. Other authors integrated crack mechanics with experiments to evaluate the strength of joints with numerous defects [8].

The development of effective simulation processes, such as the analysis of finite elements (FEM), enables the cost-effective prediction of residual stresses in large welded joints. For example, the authors of [7] present a framework for the simulation of residual stresses experimentally validated using X-ray diffraction technology. CWM is also used in additive processes, which introduces new challenges, as presented in “Approaches in computational welding mechanics applied to additive manufacturing: Review and outlook” [9]. In addition, an analysis of the fatigue of welded structures integrating CWM is important, as illustrated in [10], where the heat effects of the welding process are considered. The current investigations focus on a numerical definition and experimental validation of the welded joint fracture toughness, as shown in [11], using an analysis of finite elements in Ansys Workbench for the calculation of integral J and comparison with the test results.

Other works, such as [8], include tests of the behavior of welds with multiple defects, combining tension tests with numerical simulations, which is consistent with norms, including EN ISO 5817 [12]. Study [13] presents a general review of fracture mechanics applied to the assessment of damage to welds. A key example is [14], where a fatigue model based on FEM and linear elastic fracture mechanics was calibrated with experimental data. Quality assessment is based on geometric parameters such as the weld depth, radius of the weld tip, or axial inconsistency, which correspond to the multiple variant approach. This article compares different systems of weld quality, e.g., [12,15], showing how different quality classes affect fatigue strength, which reflects the authors’ approach to the basic concept of this study.

The role of AI in NDT should also be mentioned, as illustrated in [16]. This study discussed the integration of AI with X-ray imaging for the automatic detection of defects. In some cases, this method can reach up to 99.5% accuracy, which may be the future direction for the approach to welded joint assessment.

The authors also emphasize that, at this stage, their considerations in the methodology of the multiple criteria assessment of welded joints are theoretical concepts, and their validation on real welds is the effect of these concepts. In the following works, the authors will present examples of the validation of these solutions on real welds, performed using different methods for different minerals, to provide the basis for verifying the constructed model and evaluating the proposed assessment method’s adequacy.

## 3. Components of Welded Joint Assessment Quality

This section presents a method for assessing welded joint quality through its examination and analysis of the phenomena accompanying this process. To build such a model, it was necessary to determine a set of significant quality features *Z = X_i_*, *I =* 1, 2, …, *p*, which was divided into n disjunctive subsets *Z*_1_, *Z*_2_, *…*, *Z_n_* meeting the following dependence:

(9) $$Z_{i}\cap Z_{j}=Ø\;\mathrm{for}\;I\neq j;\;Z(t)=Z_{1}(t)\cup Z_{2}(t)\cup \ldots \cup Z_{n}(t)$$

Each of the *n*-th subsets *Z_i_*, where *i* = 1, 2, …, *n*, is a component of the set describing a welded joint quality [17,18]. Thus, a general shape of the model can be expressed by the following dependence:

(10) $$\begin{array}{l} Z_{1}(t)=\{X_{1}(t),\ldots ,X_{k_{1}}(t)\}\\ Z_{2}(t)=\{X_{k_{1}+1}(t),\ldots ,X_{k_{2}}(t)\}\\ Z_{3}(t)=\{X_{k_{2}+1}(t),\ldots ,X_{k_{3}}(t)\}\\ L\\ Z_{n}(t)=\{X_{k_{n}-r}(t),\ldots ,X_{k_{n}-1}(t),X_{k_{n}}(t)\} \end{array}$$

where:

*K_n_ = p*; *n*≤ *p*; *k*, *n*, *r*, *p*∈*N*;
*Z_i_*: subsets of characteristics describing the quality of the subsets, including elements of the *E_i_* system;
*E_i_*: quality subsets of the weld assessment;
*X_i_*: a set of characteristics that comprehensively describe the weld quality, *i =* 1, 2, …, *p*,
*i* = {1 < … < *k*_1_ < *k*_1_ + 1 < … < *k*_2_ < *k*_2_ + 1 < … < *k_n_* − *r* < … < *k_n_* − 1 < *k_n_* = *p*}.

In this study, it is assumed that the assessment of the welded joint quality is based on an analysis of four basic subsets representing the following:

- Analysis of the welding process parameters;
- Evaluation of the welded joint microstructure;
- Microscopic mechanical properties of the weld;
- Macroscopic mechanical properties of the weld.

In connection with the above, a part of the assessment model that relates to the quality of the weld (*Z_k_*) can be described by the following dependence:

*Z_k_*(*t*) = *Z*_1_(*t*) + *Z*_2_(*t*) + *Z*_3_(*t*) + *Z*_4_(*t*) (11)

where:

*Z*_1_(*t*) = *X*_1_(*t*);
*Z*_2_(*t*) = *X*_2_(*t*)+ *X*_3_(*t*)+ *X*_4_(*t*)+ *X*_5_(*t*);
*Z*_3_(*t*) = *X*_6_(*t*)+ *X*_7_(*t*) +*X*_8_(*t*);
*Z*_4_(*t*) = *X*_9_(*t*)+ *X*_10_(*t*).

Accordingly, the following can be established:

*Z_k_*(*t*) *=* [X_1_(t)]+ *+*[X_2_(t) + X_3_(t) + X_4_(t) + X_5_(t)] *+* [X_6_(t) + X_7_(t) + X_8_(t)] *+* [X_9_(t) + X_10_(t)] (12)

The quality of the welded joint described by dependence (12) is a component of its quality assessment.

To provide a comprehensive description of the weld quality, we introduce the following characteristics:

*X*_1_: rate of cooling;
*X*_2_: formation of inclusions;
*X*_3_: parameters of austenite grain;
*X*_4_: phase charts;
*X*_5_: microstructure;
*X*_6_: local strain;
*X*_7_: remaining stress;
*X*_8_: mechanical properties of the weld areas;
*X*_9_: crack resistance;
*X*_10_: bending resistance.

Currently, studies concerning the *X*_9_ and *X*_10_ features are often considered in reference to road accidents, focusing on the vehicle structure’s strength, which is crucial for the safety of driving [19].

At the same time, it must be noted that many of the identified features have a complex character and are often described similarly or expressed subjectively.

Each feature is provided with a criterion whose argument is a given feature. The identified criteria refer to different characteristics of the welding process and the weld. These parameters can be measured using different kinds of measurements and research methods, which are not always feasible. Therefore, the proposed assessment needs to enable the evaluation of the weld despite the application of only a subset of the listed criteria, which is very important from the point of view of engineering practice.

We modeled the assessment system as a multiple-criteria-based decision-making system, SMART [20].

The SMART method uses three types of criteria: MINSIMP, where the most desirable value is the smallest one; MAXSIMP, where the most desirable value is the largest one; and MAXINV, where the most desirable value is the largest possible one. The domain of each criterion is determined based on the upper and lower range of variability of the criterion argument. It is assumed that the range of variability is divided into six intervals by introducing division points. The size of each interval increases with the distance from the optimum, according to a geometric series with a quotient equal to two.

Depending on the type of criterion, the shape is described by different functions. For the MINSIMP and MAXINV criterion, the function takes the form (13):

(13) $$f_{c}\left(a\right)=\log_{2}\left(\frac{a-a_{min}}{a_{max}-a_{min}}\cdot 64\right),$$

where:

*a_max_* represents the upper range of variability of the criterion argument;
*a_min_* represents the lower range of variability of the criterion argument;
*a* represents the value of the criterion argument for the division point;
*f_c_*(*a*) represents the value of the criterion function for the division point.

For the MAXSIMP criterion, the function is described by Formula (14):

(14) $$f_{c}(a)=\log_{2}\left(\frac{a_{max}-a}{a_{max}-a_{min}}\cdot 64\right).$$

For the MINSIMP and MAXINV criterion, the values of the criterion argument for the division points are calculated by Formula (15) and, for the MAXSIMP criterion, by Formula (16).

(15) $$a(v)=a_{min}+\left(a_{max}-a_{min}\right)\cdot \frac{2^{\nu }}{64},\nu =0,1,\ldots ,6$$

(16) $$a\left(v\right)=a_{max}-\left(a_{max}-a_{min}\right)\cdot \frac{2^{\nu }}{64},\nu =0,1,\ldots ,6$$

For the MINSIMP and MAXSIMP criteria, a scale from 4 to 10 is introduced, according to Formula (17), while for MAXINV, it is described by Formula (18):

(17) $$g=10-f_{c}(a),$$

where:

*g* represents the degree of the criterion fulfilment.

(18) $$g=4+f_{c}(a)$$

Equal weights have been introduced for each of the developed criteria.

**Figure 2:**
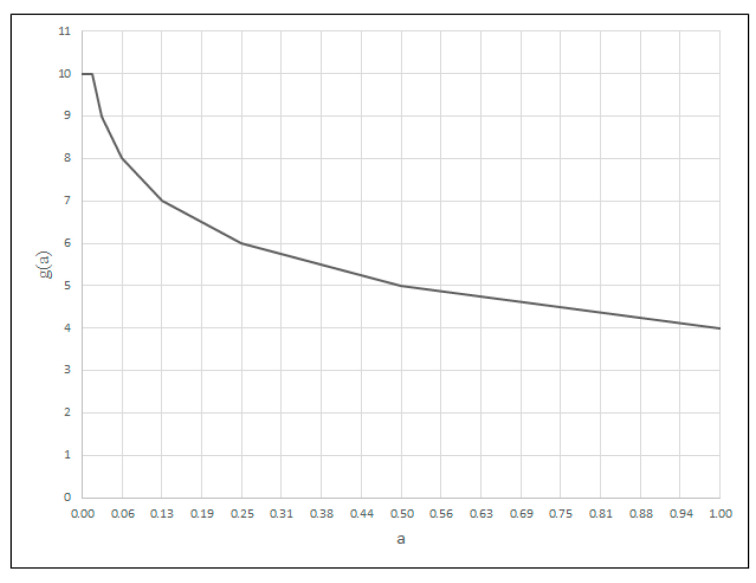
Membership function of the MINSIMP criterion.

**Figure 3:**
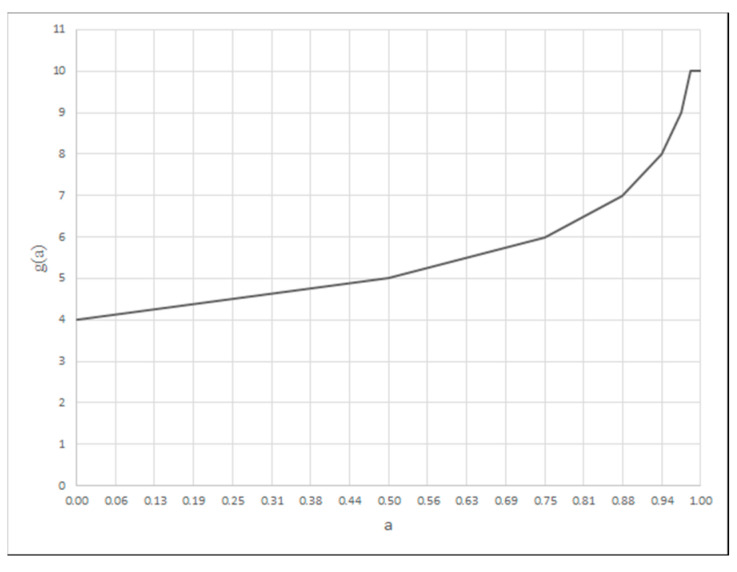
Membership function of the MAXSIMP criterion.

**Figure 4:**
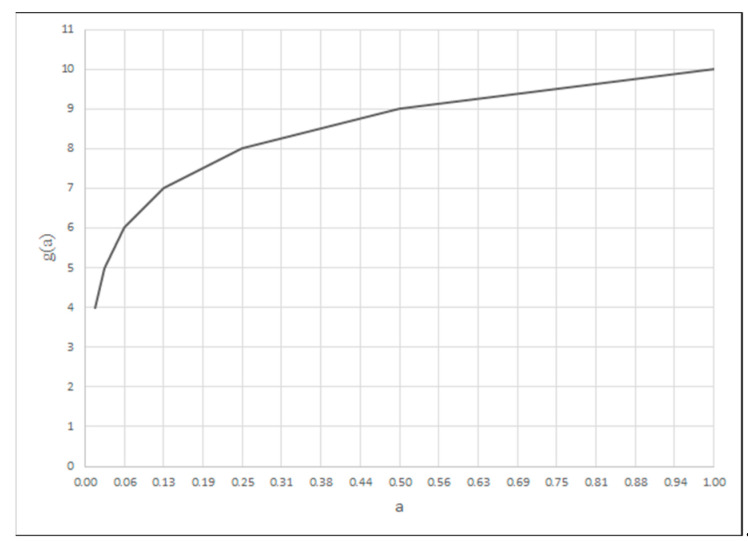
Membership function of the MAXINV criterion.

For the criterion for which the smallest value is most desirable (MINSIMP), a degree of the criterion fulfilment is modeled by the function whose shape is shown in Figure 2, calculated based on Formulas (13), (15) and (16). The criterion for which the largest value is the most desirable (MAXSIMP) is modeled as a function calculated from Formulas (14), (16) and (17) (Figure 3). In the case of the criterion for which the largest possible value is the most desirable (MAXINV), the function of the criterion fulfilment degree presented in Figure 4 is used. It is calculated from Formulas (13), (15) and (18).

A normalized weight *c* is determined for each criterion. The total rating of variant *s* is calculated according to Formula (19):

(19) $$s=\sum_{i=1}^{k}c_{i}\cdot g_{i},$$

where:

*s* represents the total rating of the variant;
*c* represents the normalized weight of the criterion;
g represents the degree of the criterion fulfilment;
*i* represents the number of the criterion.

The value of the final grade ranges from 4 to 10. This allows for the verbal interpretation of the value (Table 1).

## 4. Modeling of the Assessment Process of the Welded Joint Characteristics

### 4.1. Characteristics Describing the Welding Process Parameters

The cooling rate influences the weld microstructure, internal stresses, and fracture toughness. The criterion for this feature was modeled in MAXINV form (Figure 4). The cooling time t_n_ (from max temperature to 500 °C) in the range of 5–50 s was used as the criterion argument.

### 4.2. Characteristics Describing the Weld Microstructure

#### 4.2.1. Formation of Inclusions

This comprises the results of the research on ultrasound inclusions in the weld, which are evaluated based on the amplitude of the reflected signal (compared to the referential echo), the size of inclusion or defect (length and depth), as well as the localization of a defect in the weld. Based on this, norm [21] defines three classes of weld quality.

Class 1: the highest quality (no tolerance for larger defects): echoes of large amplitude (e.g., exceeding the referential echo by more than 20%) are usually considered acceptable. Larger size inclusions, particularly those located in critical parts of the joint, are not tolerated.

Class 2: medium quality (acceptable smaller defects): inclusions and other defects of mild amplitude and size are accepted unless they significantly affect the joint resistance.

Class 3: lower quality (higher acceptance of defects): defects of relatively high amplitude and size can be acceptable unless their location is critical for the structure’s strength.

**Figure 5:**
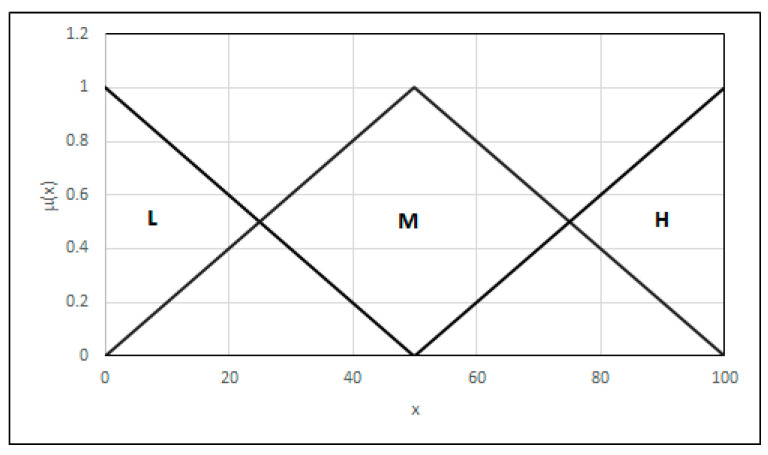
Linguistic variable—amplitude of the reflected signal.

We modeled the amplitude of a reflected signal in the form of a linguistic variable using three fuzzy sets of type L (low amplitude—L) (20), Λ (medium amplitude—M) (21), and Γ (high amplitude—H) (22), successively covering the whole domain of the initial value (Figure 5),

(20) $${FS}_{L}\left(x\right)=\left\{\begin{array}{c} 1\\ \frac{rrs-x}{rrs-rrk}\\ 0\; \end{array}\right.\begin{array}{c} \;\Leftrightarrow x\leq rrk\\ \;\;\;\;\;\;\;\;\;\;\;\;\;\Leftrightarrow rrk\leq x\leq rrs\\ \Leftrightarrow x>rrs \end{array},$$

(21) $${FS}_{\Lambda }\left(x\right)=\left\{\begin{array}{c} 0\\ \frac{x-lrs}{lrk-lrs}\\ \frac{rrs-x}{rrs-lrk} \end{array}\right.\begin{array}{c} \;\;\;\;\;\;\;\;\;\;\;\;\Leftrightarrow x\leq lrs\vee x\geq rrs\\ \;\;\;\;\Leftrightarrow lrs<x\leq lrk\\ \;\;\;\;\;\Leftrightarrow lrk<x<rrs \end{array},$$

(22) $${FS}_{\Gamma }\left(x\right)=\left\{\begin{array}{c} 0\\ \frac{x-lrs}{rrs-lrs}\\ 1\; \end{array}\right.\begin{array}{c} \;\Leftrightarrow x\leq lrs\\ \;\;\;\;\;\;\;\;\;\;\;\;\;\Leftrightarrow lrs\leq x\leq rrs\\ \;\Leftrightarrow x>rrs \end{array},$$

where:

*FS* represents the fuzzy set;
*x* represents the argument of a fuzzy set;
*lrs* represents the left range of the fuzzy set support;
*lrk* represents the left range of the fuzzy set kernel;
*rrs* represents the right range of the fuzzy set support;
*rrk* represents the right range of the fuzzy set kernel.

This partitioning is marked by {*L*_(0,0,50)_, Λ_(0,50,100)_, Γ_(50,100,100)_} as a list of fuzzy sets for which the left extreme value of the medium, the kernel, and the right extreme value of the medium are established.

Analogically, a linguistic variable was obtained for the size of the inclusion, which consists of three fuzzy sets representing a small inclusion (fuzzy set type L), a medium inclusion (fuzzy set type Λ), and a large inclusion (fuzzy set types Γ) {*L*_(0,0,50)_, Λ _(0,50,100)_, Γ_(50,100,100)_}. When the inclusion is located as an argument of the linguistic variable, its critical distance from the joint critical areas is considered. However, contrary to the inclusion size and the amplitude size of a reflected signal, the shortest distance from the critical surfaces is the worst; hence, in this case, the distance transformed using Equation (23) is an argument of the linguistic variable.

(23) $$d’=d_{max}-d,$$

where:

*d’* represents the transformed distance,
*d_max_* represents the maximum distance,
*d* represents prior to the transformation distance.

Following the transformation, the linguistic variable was modeled just like the inclusion size. The linguistic variables, inclusion size, and localization were ultimately modeled, as shown in Figure 5.

To calculate the final size describing the formation of inclusions, we accepted, for each parameter, the sum of the products of the fuzzy set orders (0,1,2) and the value of the membership function (24):

(24) $$v_{c}=\sum_{i=0}^{n}i\cdot \mu_{FSi}(v),$$

where:

*v_c_* represents the value of the criterion argument,
*FS_i_* represents the *i*-th fuzzy set,
*μ_FSi_*(*v*) represents the value of the membership function and the value *v* of the *i*-th fuzzy set.

Thus, the descriptive value of each parameter will be in a (0–3) range. In this way, the argument of the criterion relating to the formation of inclusions will be a value within a (0–27) range (product of the values for each parameter). The larger the argument’s value, the worse the weldability. Hence, this is the MINSIMP criterion: the best lower value (Figure 2).

#### 4.2.2. Parameters of Austenite Grain

Norm [22] describes a method for assessing and determining the value of ferrite or austenite grain in steel and other metals using optical microscopy. The ASTM number is used to evaluate the grain size (ASTM E112: standard for measurement of grain value in metal materials) in a 0–14 range, where a lower number represents more tiny grains. This is desirable, as more tiny grains positively impact grain strength, plasticity, fatigue, abrasion, and fracture toughness. Therefore, we modeled a criterion in the form of the MINSIMP (Figure 2) for a (0–14) range of arguments.

#### 4.2.3. Presence of Each Microstructural Phase in the Joint

**Figure 6:**
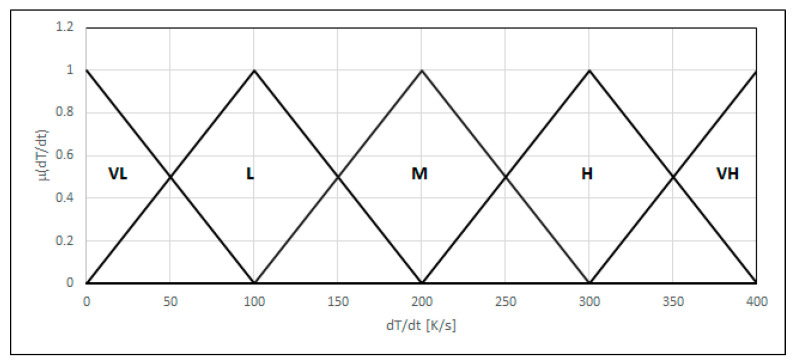
The linguistic variable: cooling rate.

This feature was evaluated using an analysis of the CTPc-S phase diagram. The CTPc-S diagram presents a dependence between the time of cooling and the temperature characteristic of the phase transitions in steels or other metals during welding. The diagram makes it possible to determine which structural phases will be present in a weld depending on the rate of cooling. This can be ferrite, bainite, martensite, or austenite. Since the diagram is developed empirically, and determination of the cooling speed can be problematic, we modeled the cooling rates as a linguistic variable in the form of five fuzzy sets (very small, small, medium, large, and very large cooling speed) {*L*_(0,0,100)_, Λ_(0,100,200)_, Λ_(100,200,300)_, Λ_(200,300,400)_, Γ_(300,400,400)_}. The first set of type L, the last set Γ, and the medium Λ covering the cooling rate dT/dt in a 0–400 range (Figure 6) are considered in the diagram.

Each fuzzy set is assigned the number of phases in the form of a fuzzy set type Λ. Next, the number of phases in each weld is provided using fuzzification of the cooling rate value, the MIN–MAX inference, and defuzzification using the height assessment method (25).

(25) $$y=\frac{\sum_{FS}\mu (y)\cdot y}{\sum_{FS}\mu (y)},$$

where:

*y* represents the crisp value,
*FS* represents the fuzzy set,
*μ*(*y*) represents the value of the function of membership of the *y* value in the *FS* fuzzy set.

We modeled a criterion in the form of MAXINV (Figure 4) for an argument that is a percentage share of desirable phases in the weld (e.g., bainite and bainitic ferrite in low alloy and high resistant steels) for a 0–100% range of arguments (100% corresponds to the highest possible concentration of these phases in the weld; it is not 100% of these phases’ content).

#### 4.2.4. Microstructure

**Figure 7:**
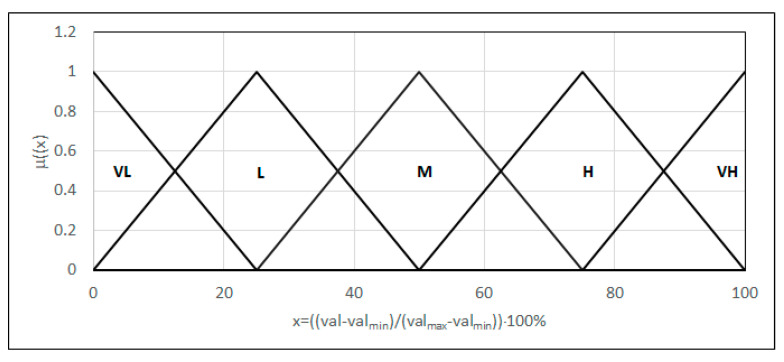
Forms of the linguistic variables—microstructure and local strain parameters. Here, val represents the parameter value, val_min_ represents the minimum value of the parameter, and val_max_ represents the maximum value of the parameter.

We assessed the structure through evaluation of the amount of austenite in the weld microstructure (α), the amount of austenite in the heat-affected zone (α_w_), the amount of austenite in the weld (α_a_), the temperature of the martensitic transition (*M_s_*), and the temperature of the bainite transition (*B_s_*). We modeled each of the quantities in the form of linguistic variable as five fuzzy sets (Figure 7) {L_(0,0,25)_, Λ_(0,25,50)_, Λ_(25,50,75)_, Λ_(50,75,100)_, Γ_(75,100,100)_}; however, in this case, the range of each argument was assumed to be a 0–100 range, where 0 corresponds to the smallest and 100 to the largest possible value of the attribute. The same modeling was accepted for the whole assessment of the microstructure.

The application of a set of rules formulated based on norms [23,24], the MIN–MAX inference, and defuzzification via the height assessment method (25) allow for obtaining a clear value of the weld microstructure assessment. The obtained value is an argument of the MAXSIMP criterion (Figure 3).

### 4.3. Features Describing the Microscopic Properties of a Mechanical Weld

#### 4.3.1. Local Strains

Assessment of local strains should be performed by evaluating the plastic strain (e_p_), elastic strain (e_e_), thermal deformation (e_c_), and strain in the heat-affected zone (e_ph_). As with the previous items, we modeled each quantity in the form of a linguistic variable as five fuzzy sets (Figure 7) {L_(0,0,25)_, Λ_(0,25,50)_, Λ_(25,50,75)_, Λ_(50,75,100)_, Γ_(75,100,100)_}. The same modeling was accepted for the entire assessment of the local strains. The application of a base of rules formulated from the directives of the norm [23], the MIN–MAX inference, and defuzzification via the height assessment method, described by dependence (17), makes it possible to obtain the weld quality assessment value in terms of the local strains. The value obtained is an argument of the MAXSIMP criterion (Figure 3).

#### 4.3.2. The Residual Stress

**Figure 8:**
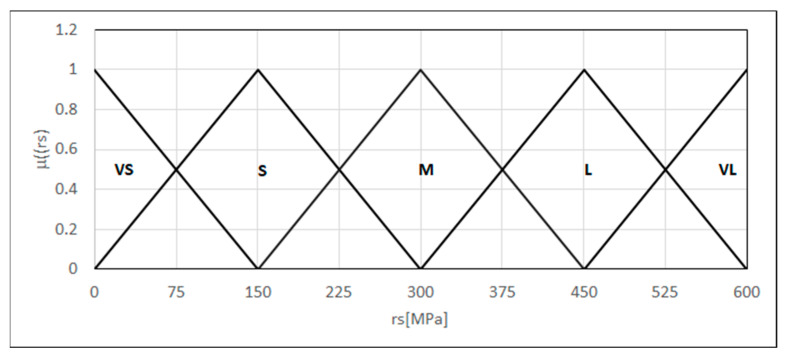
Forms of the linguistic variable—residual stress.

We formulated a criterion for the residual stresses based on the measurement results using (XRD) X-ray diffraction, according to the norm [25]. The considered norm does not provide detailed assessment numerical values because acceptable levels of the remaining stresses can differ depending on the material type, its application, and branch standards. Therefore, we modeled a feature in the form of a linguistic variable as five fuzzy sets (very small, small, medium, large, and very large remaining stresses) {L_(0,0,150)_, Λ_(0,150,300)_, Λ_(150,300,450)_, Λ_(300,450,600)_, Γ_(450,600,600)_}. The variability range of the argument is from 0 to 600 MPa (Figure 8).

For the considered value of residual stresses, summing the values of the products of the affinity degree for all fuzzy sets and their successive numbers provides the values of the criterion arguments in a 0 to 4 range. Using this defined argument, the MINSIMP criterion is formulated (17).

#### 4.3.3. Mechanical Properties of the Welded Joint Areas

We use toughness (Vickers method), tensile, impact, and micro-toughness tests to evaluate the mechanical properties of the joint areas.

**Figure 9:**
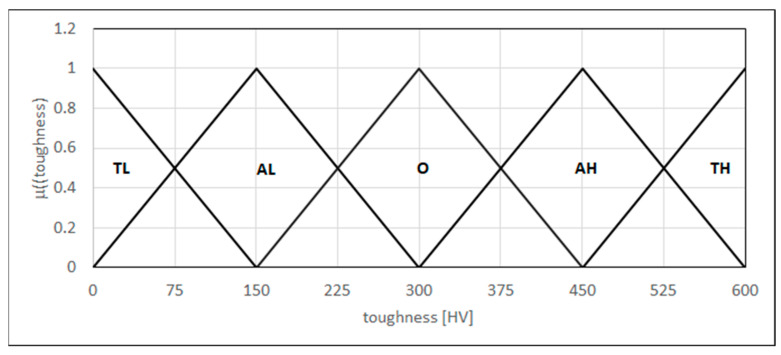
Forms of the linguistic variable—toughness. TL: too low toughness; AL: acceptably low toughness; O: optimal toughness; AH: acceptably high toughness; TH: too high toughness.

The toughness test measures the toughness along the weld cross-section, including the parent metal, the heat-affected zone (HAZ), and the weld itself. Measurement along the lines allows identification of the toughness differences that can occur in three areas. We modeled the toughness as a linguistic variable in the form of {L_(0,0,150)_, Λ_(0,150,300)_, Λ_(150,300,450)_, Λ_(300,450,600)_, Γ_(450,600,600)_} in the range of 0–600HV, where the fuzzy sets represent too low toughness, acceptably low toughness, optimal toughness, acceptably high toughness, and too high toughness, respectively (Figure 9).

#### 4.3.4. Tensile Test

**Figure 10:**
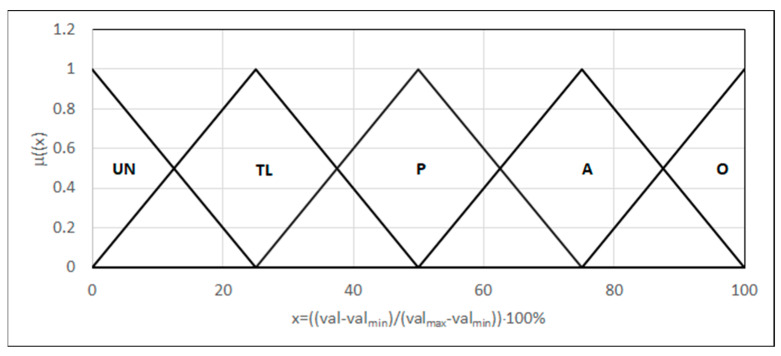
The form of the linguistic variables: tensile strength and relative extending. val: the parameter value; valmin: the minimum value of the parameter; valmax: the maximum value of the parameter.

Three tensile strength parameters were evaluated (Rm), as well as the relative extending (A) and the place of rupture occurrence. Linguistic variables in the form {L_(0,0,25)_, Λ_(0,25,50)_, Λ_(25,50,75)_, Λ_(50,75,100)_, Γ_(75,100,100)_} represent the tensile strength and relative extending, for ranges defined by a respective norm, adjusted to the range of 0–100: unacceptable values, too low values, permitted values, acceptable values, and optimal values (Figure 10).

**Figure 11:**
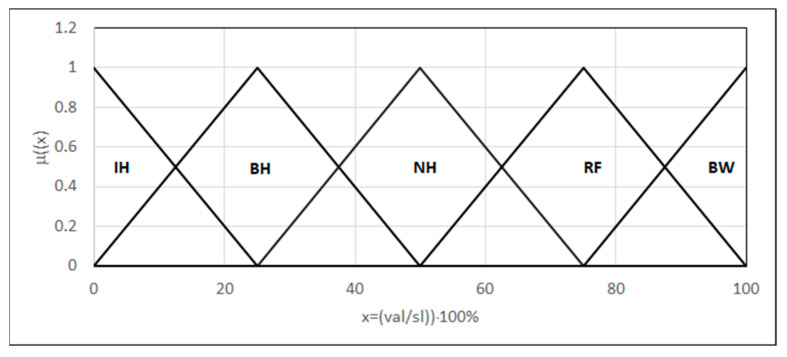
Forms of the linguistic variables—the place of rupture. val: place of rupture; sl: the sample length.

For the rupture occurrence location, we introduced a linguistic variable distance from the HAZ in the form {*L*_(0,0,25)_, Λ_(0,25,50)_, Λ_(25,50,75)_, Λ_(50,75,100)_, Γ_(75,100,100)_} modeled using fuzzy sets: ruptures in the HAZ, at the border of the HAZ, near the HAZ, relatively far from the HAZ, and beyond the weld. The argument range is the specimen dimension transformed to 0–100 (Figure 11).

**Figure 12:**
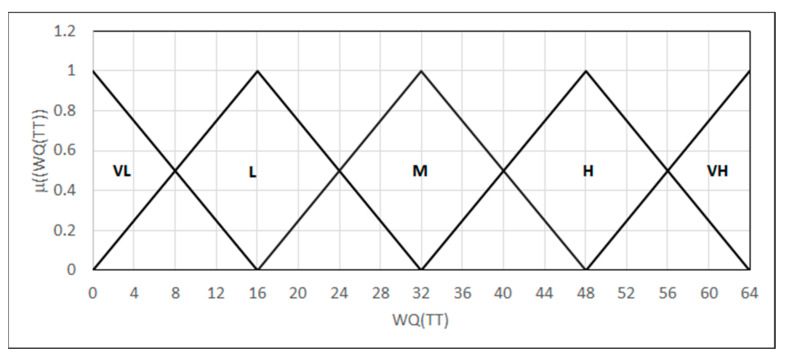
Forms of the linguistic variables—WQ(TT): the weld quality in terms of the tensile test.

The weld quality in terms of each parameter is calculated as the value of the product of the function of membership in each fuzzy set and the number of fuzzy sets. The entire result of the tensile test, calculated as the product of values (0–64), is then modeled in the form of linguistic variables {*L*_(0,0,16)_, Λ_(0,16,32)_, Λ_(16,32,48)_, Λ_(32,48,64)_, Γ_(48,64,64)_} describing the weld quality in terms of the tensile test (26) (Figure 12).

(26) $$WQ(TT)=\prod_{Rm,\;A,Sr}\sum_{i=0}^{4}i\cdot \mu_{{FS}_{i}}(x),$$

*WQ*(*TT*) represents the weld quality in terms of the tensile test;
*Rm* represents the tensile strength;
*A* represents the relative extending;
*Sr* represents the place of the specimen rupture; *i* represents the number of fuzzy sets;
*x* represents the argument of the fuzzy variable;
*μ_FSi_*(*x*) represents the value of the membership function of the *i*-th fuzzy set.

#### 4.3.5. Impact Test (Charpy V-Notch)

**Figure 13:**
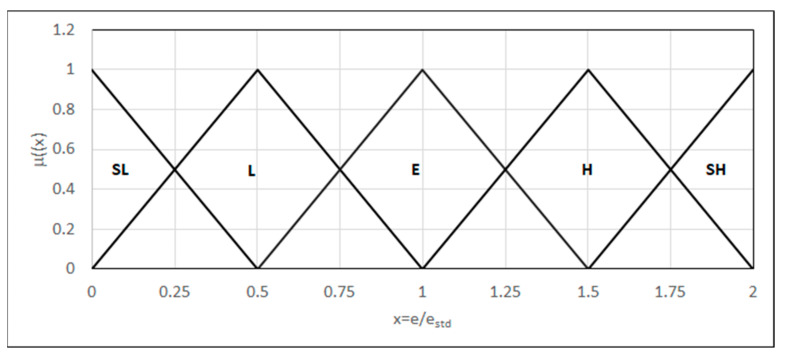
Forms of the linguistic variables—e: the energy absorbed by the material; e_std_: the energy set out in the standard.

In this case, the parameters considered are the energy absorbed by the material and the character of the rupture. We modeled the energy absorbed by the material as a linguistic variable using five fuzzy sets {L_(0,0,0.5)_, Λ_(0,0.5,1)_, Λ_(0.5,1,1.5)_, Λ_(1,1.5,2)_, Γ_(1.5,2,2)_}: significantly higher, higher, equal, lower, and significantly lower energy than those set out in the relevant norm, respectively. The parameters variability range and localization within this range of the fuzzy sets are defined depending on the value set out in the norm (Figure 13).

**Figure 14:**
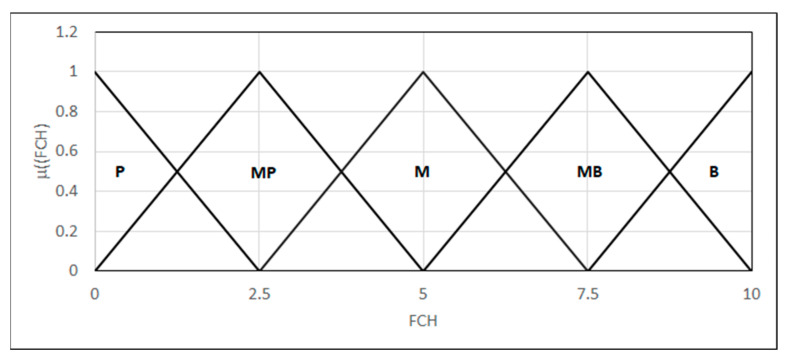
Forms of the linguistic variables—FCH: the fracture character.

As for the fracture character, we introduced a 0–10 scale, where 0 represents a plastic fracture and 10 represents a brittle fracture. In this range, the fracture character can be described by a linguistic variable modeled using five fuzzy sets {*L*_(0,0,2.5)_, Λ_(0,2.5,5)_, Λ_(2.5,5,7.5)_, Λ_(5,7.5,10)_, Γ_(7.5,10,10)_}: plastic fracture, a mixed fracture with a predominance of plastic fracture, mixed, mixed with a predominance of brittle, and brittle fracture, respectively (Figure 14).

#### 4.3.6. Micro-Toughness Assessment

**Figure 15:**
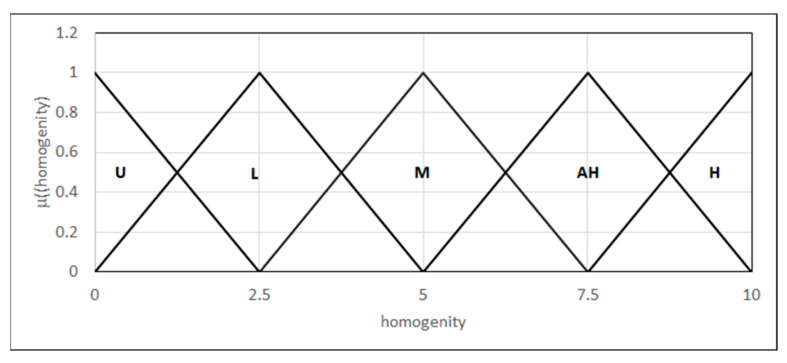
Forms of the linguistic variables—homogeneity.

This involves measuring the toughness on a micro-scale in different zones of the welded joint, that is, the joint, the HAZ, and the parent material. It is used for the analysis of microstructural properties at the local level and allows the identification of the differences in toughness, which can indicate changes in the joint microstructure and its quality. We modeled the differences in the microstructure of particular phases by introducing a 0–10 scale, where 0 represents the unsatisfying homogeneity of the joint and 10 represents high homogeneity. We formulated a linguistic variable in the range {L_(0,0,2.5)_, Λ_(0,2.5,5)_, Λ_(2.5,5,7.5)_, Λ_(5,7.5,10)_, Γ_(7.5,10,10)_} using fuzzy sets: unsatisfactory homogeneity, low homogeneity, medium homogeneity, acceptably high, and high homogeneity, respectively (Figure 15).

**Figure 16:**
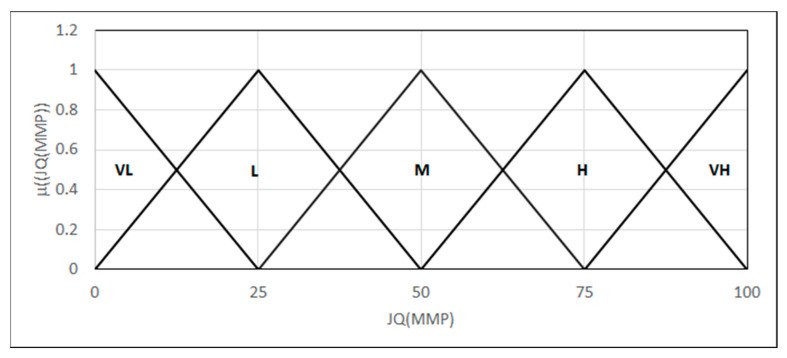
Forms of the linguistic variables—JQ(MMP): the quality of the joint in terms of the microscopic mechanical properties.

Finally, we built a base of inference rules for the linguistic variables provided in each test and introduced a linguistic variable quality of the joint in terms of the microscopic mechanical properties of the particular weld areas {*L*_(0,0,25)_, Λ_(0,25,50)_, Λ_(25,50,75)_, Λ_(50,75,100)_, Γ_(75,100,100)_} (Figure 16).

The resultant quality value for a 0–100 scale is obtained using the MIN–MAX inference, with defuzzification via a height operator (25). A criterion is introduced when treating the obtained value as an argument of the MAXSIMP (Figure 3).

### 4.4. Features Describing the Macroscopic Mechanical Properties of the Weld

#### 4.4.1. Crack Resistance

**Figure 17:**
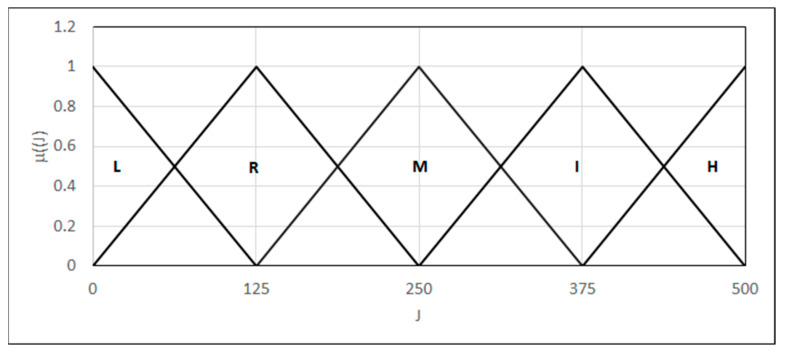
Forms of the linguistic variables—critical coefficient J.

We introduced a criterion based on an integral method, J, which measures the material’s resistance to crack initiation and propagation. The scale of crack resistance assessment is based on the values of the critical coefficient J (J_IC), which defines the energy needed to initiate a crack in a unit of the surface. We built a grading scale based on norm [26] directives by introducing five, instead of three, degrees, modeled by fuzzy sets representing low, reduced, medium, increased, and high crack resistance, respectively {L_(0,0,125)_, Λ_(0,125,250)_, Λ_(125,250,375)_, Λ_(250,375,500)_, Γ_(375,500,500)_}, accepting the parameter range to be 0–500 [kJ/m^2^] (Figure 17).

The final value is calculated as a sum of the values of the affinity function products and the number of fuzzy sets for the analyzed value of the J coefficient. So, the created value is an argument of the MAXSIMP criterion formulated for values in the 0–4 range.

#### 4.4.2. Bending Resistance

**Figure 18:**
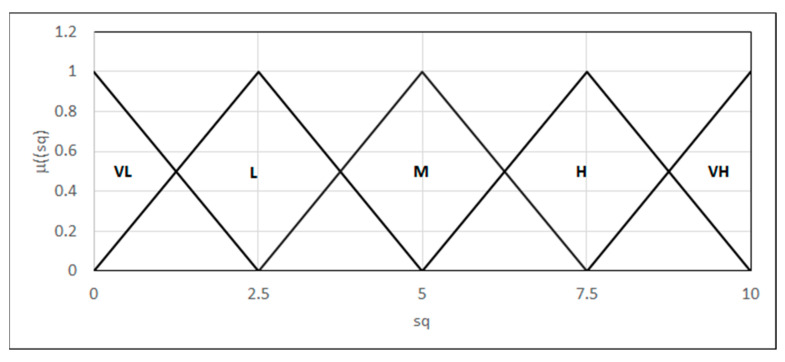
Forms of the linguistic variables—the quality of the surface: sq.

Two parameters are evaluated: the quality of the specimen surface and the weld strength. We introduced a subjective grading scale, 0–10, for each parameter to be formulated in reference to the values set out in norms [27]. Each parameter was modeled in the form of a linguistic variable {L_(0,0,2.5)_, Λ_(0,2.5,5)_, Λ_(2.5,5,7.5)_, Λ_(5,7.5,10)_, Γ_(7.5,10,10)_}, defining fuzzy sets for linguistic variable “the quality of the specimen surface” (Figure 18):

- Very low surface quality: the presence of large cracks, delaminations, or other defects after bending;
- Low surface quality: the presence of small surface cracks whose lengths or widths do not exceed the acceptable boundaries defined by the norms;
- Medium surface quality: there may be small surface cracks, the length or width of which is within the permissible limits specified by the standards;
- High surface quality;
- Very high surface quality: no cracks or delaminations after the bending test.

**Figure 19:**
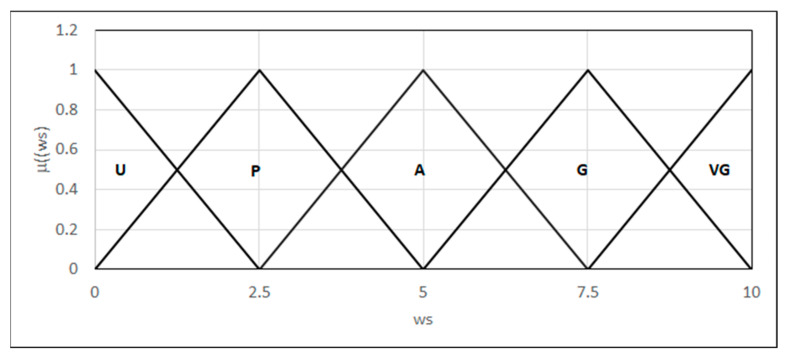
Forms of the linguistic variable—the weld strength: ws.

We also defined the linguistic variable “weld strength” (Figure 19):

- Unacceptable strength: the weld exhibits low ductility and susceptibility to brittle cracking;
- Permissible strength;
- Acceptable strength: small defects that do not affect the weld strength;
- Good strength;
- Very good strength: the joint exhibits high ductility and good adhesion to the parent material.

The weld quality in terms of each parameter is calculated as a product of the value of the function of membership in each fuzzy set and the fuzzy set number, whereas the total result of the bending test is calculated as the product of the values obtained for each parameter (27).

(27) $$WQ(BR)=\prod_{sq,ws}\sum_{i=0}^{4}i\cdot \mu_{{FS}_{i}}(x),$$

where:

*WQ*(*BR*) represents the weld quality in terms of the bending resistance; sq represents the surface quality;
*ws* represents the weld strength;
*i* represents the number of fuzzy sets;
*x* represents the argument of the fuzzy variable;
*μ_FSi_*(*x*) represents the value of the membership function of the *i*-th fuzzy set.

The obtained value is an argument of the MAXSIMP criterion, formulated for values within the (0,16) range.

## 5. Conclusions

The proposed modeling enables the creation of a multiple-criteria-based system for assessing a welded joint’s quality to allow its evaluation, according to Table 1. Thus, it enables a qualitative assessment of the welded joint and the clear quantification of its value according to the accepted scale. The value of this assessment depends on the weight assigned to the features. Their significance, however, can change depending on the criteria accepted. Therefore, the presented assessment method is universal from a given point of view and can be applied in evaluating widely understood sociotechnical systems of <C-M-O> (human–machine–environment) type or their component systems.

The next step in the authors’ research is validating the proposed method. Models will be built to assess the quality of welded joints made of different materials using different methods, considering the set criteria and the significance of particular features.

## Figures and Tables

**Table 1:** Interpretation of the variant’s total rating value.

Total Rating Values	Interpretation
10	Ideal
9	Very good
8	Good
7	Poor
6	Very poor
5	Bad
4	Very bad

## Data Availability

The original contributions presented in this study are included in the article. Further inquiries can be directed to the corresponding author.
